# A community-based participatory approach to delivering the *Dealing with Dementia* program to Black caregivers

**DOI:** 10.3389/fpubh.2025.1676590

**Published:** 2025-10-21

**Authors:** Karah Alexander, Fayron Epps, Jacque Thornton, Mia Chester, Toni Miles

**Affiliations:** ^1^Nell Hodgson Woodruff School of Nursing, Emory University, Atlanta, GA, United States; ^2^UT Health San Antonio, School of Nursing, San Antonio, TX, United States; ^3^Alter Dementia, Atlanta, GA, United States; ^4^Sage Navigator Inc., East Point, GA, United States; ^5^College of Public Health, University of Georgia, Athens, GA, United States

**Keywords:** dementia, Alter, community participatory research, caregivers, Georgia, churches

## Abstract

**Background:**

Alter is a community-based program created to address gaps in dementia awareness and caregiving support/resources for Black and faith communities. The Rosalynn Carter Institute for Caregivers (RCI) collaborated with Alter to adapt the existing *Dealing with Dementia* (DWD) program [DWD_Alter_] to better reach and meet the needs of Black families through relevant dementia education and practical caregiving strategies.

**Context:**

Faith-based partnerships were identified as a trusted place to start to deliver the pilot DWD_Alter_ Program. Four focus groups were conducted with Black faith leaders, their congregants who identified as caregivers, and other community members to inform the adaptation of the program. RCI’s original agency-based DWD model was modified using the principles of Community-Based Participatory Research.

**Programmatic elements:**

Input from the focus groups shaped the DWD_Alter_ protocol, which included: (a) facilitator “toolkit,” (b) program assessments, (c) procedure manual, and (d) a community recruitment plan. To test the pilot DWD_Alter_, 22 Black community members were trained as facilitators, including 10 (45%) from rural Georgia communities. Between June and August 2024, 15 facilitators conducted 27 DWD_Alter_ sessions using these new materials. Outcomes of this pilot initiative include an assessment of participant demographics and program satisfaction (acceptability). Two hundred and sixty-four persons attended the sessions, 95% of whom identified as Black or African American. Seventy-nine percent were family caregivers for individuals living with dementia, and 83% strongly agreed that the program met their caregiving needs. Evaluation of program effectiveness, specifically changes in caregiving self-efficacy and dementia knowledge before and after participation, will be conducted at a later time.

**Discussion:**

While program acceptability was high, delivery challenges included limited facilitator availability, a 2.5-month grant period, and outreach barriers in Black rural communities. Following the pilot field test, a sustainability plan was co-developed with community facilitators to support continued implementation and ensure that resources spent on DWD_Alter_ were not lost.

## Introduction

Caregiving is a nearly universal experience, but the intersection of race, culture, and social inequities can make it especially demanding for Black dementia caregivers. Black caregivers face not only the general challenges of caregiving but also unique stressors shaped by cultural norms, socioeconomic barriers, and institutionalized disparities (1). For example, research indicates that compared to White caregivers, Black caregivers are more likely to provide intensive care, over 40 h each week (54% vs. 39%), and are also more often caring for someone living with dementia living below the federal poverty line (32% vs. 12%) (2). They also report higher use of supportive services than White caregivers (33% vs. 25%) (2). Such disparities extend beyond care hours and service use—they also include inequities in access to healthcare, financial resources, and reliable social support. While the literature on dementia caregiving is extensive, less is known about the needs of caregivers in rural communities, as most research reflects urban settings.

Rural caregivers of persons living with dementia can face unique challenges, including limited access to healthcare, transportation barriers, and a shortage of affordable support services (3). Even when services exist, geographic isolation, economic disadvantage, and weak community infrastructure create additional barriers (3). Demographic trends further heighten these challenges. With younger adults leaving for education and employment, rural areas are aging more rapidly than urban ones (3). In the United States (U.S.), 17.5% of rural residents are aged 65 years and older compared with 14.9% nationwide, and nearly three-quarters of rural older adults live in the South (4). As this population ages, the risk of developing dementia and demand for caregiving will continue to rise (5). Currently, about one in five caregivers (20%) live in rural communities (6). Yet, within the already limited research on rural dementia caregiving, the specific needs of Black rural caregivers remain especially overlooked (3). According to the 2020 Census, approximately 4.5 million Black individuals lived in rural areas of the U.S., accounting for 7.4% of the rural population. Although this number has declined in recent years, the proportion of Black rural residents remains highest in the South (4). Research further indicates that Black caregivers represent about 6% of reported rural caregivers, yet they have higher odds of experiencing unmet needs in rural areas compared with their White counterparts (7). Given that Black caregivers often encounter racial and ethnic disparities, when combined with the structural disadvantages of rural environments, these overlapping burdens place rural Black caregivers at the intersection of multiple inequities, underscoring the need for targeted resources and interventions.

Based in Americus, Georgia, for nearly 10 years, the Rosalynn Carter Institute for Caregivers (RCI) has been a leader in providing resources to dementia caregivers through the delivery of its *Dealing with Dementia* (DWD) program (8). DWD was developed out of a recognized need for family caregivers to have practical guidance when caring for persons living with dementia. As part of the DWD program, caregivers receive a comprehensive guide with over 300 pages of detailed information within 34 chapters grouped by the following five key sections: (1) Understanding Dementia, (2) General Caregiving Tips, (3) Dealing with Behavioral Issues, (4) Self-Care, and (5) Resources. The guide also includes fillable handouts on emergency information, hospital-to-home records, personal health records, self-care strategies, medication management, a handout for searchers, and problem-solving that caregivers are encouraged to fill out and keep handy. To help optimize use of the guide, caregivers participate in a workshop that details the key topics and helps them understand dementia, manage problem behaviors, and take better care of themselves. DWD is a data-driven program that has served more than 7,500 caregivers from 41 states and Washington, D.C. Participants in DWD have demonstrated an increase in dementia knowledge and confidence in their ability to provide care (8).

## Context

Providing free and accessible dementia education through Black faith communities is a promising strategy for addressing caregiving disparities. In 2024, the Rosalyn Carter Institute for Caregivers (RCI), partnered with Alter, a nurse-led, community-based program focused on building resources and awareness around dementia in Black and faith communities (9). This collaboration sought to address the challenges faced by Black caregivers by engaging community members to adapt the existing, evidence-based *Dealing with Dementia* (DWD) program [DWD_Alter_] and extend its reach and relevance to Black caregivers in Georgia, with particular attention to reaching those in rural communities. The primary intent of the collaboration was to engage trusted community-based partners in delivering relevant resources and education directly into the hands of Black dementia caregivers who need them the most.

In this Community Case Study, the programmatic elements, outcomes, implications, and limitations of the pilot DWD_Alter_ Program are described, along with a plan for its sustainability. Outcomes of this pilot program include an evaluation of participant demographics and program satisfaction (acceptability). Assessment of program effectiveness, specifically changes in caregiving self-efficacy and dementia knowledge before and after participation, is currently underway in collaboration with RCI’s Data Manager and analysis team.

**Figure 1:**
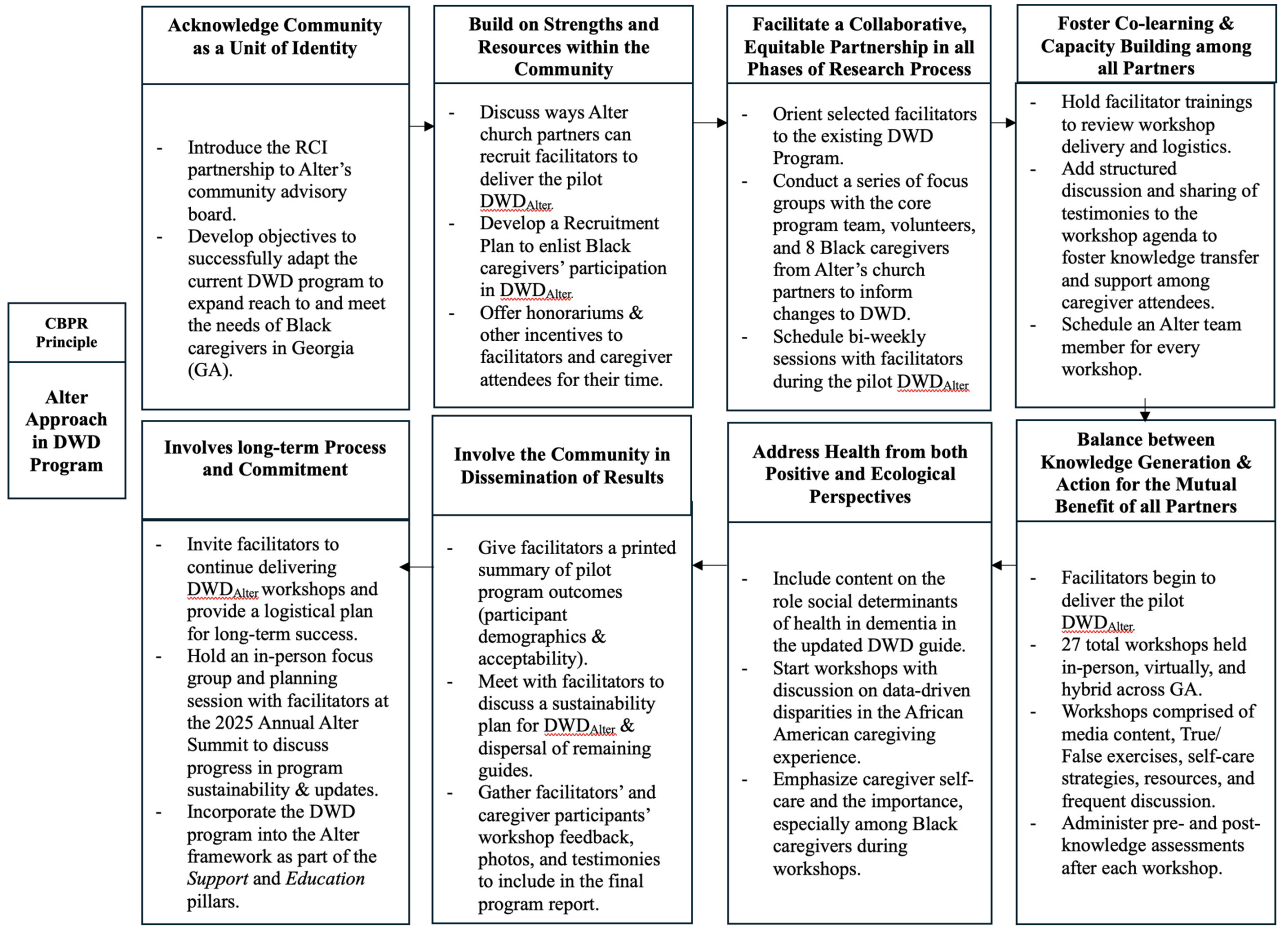
A CBPR-driven approach to *Dealing with Dementia*: a guide for Black caregivers (DWD).

In general, Alter uses a Community-Based Participatory Research (CBPR) approach when conducting research and addressing dementia-related disparities and inequities in Black communities. CBPR is a partnership approach that equitably involves community members, researchers, organizations, and other stakeholders in research and practice processes, and recognizes the unique strengths that each brings (21). CBPR aims to combine knowledge and action to create positive and lasting social change. CBPR has become a common research approach in public health, medicine, and nursing (21). To successfully deliver the pilot DWD_Alter_ to Black caregivers and evaluate its outcomes, RCI’s existing DWD program and protocol were adapted to align with the 8 key principles of CBPR (10, 21). Figure 1 depicts how each CBPR principle was carried out during DWD_Alter_. The following sections provide detailed descriptions of how these principles were intentionally implemented.

## Programmatic elements of the adapted DWD program

To begin revising the DWD program, the core program team was first oriented to the existing DWD program through a 1.5-day training with an RCI DWD Program Specialist. We then discussed the RCI partnership with the Alter community advisory board, composed of faith leaders, community members, caregivers of persons living with dementia, and individuals living with dementia. After addressing the concerns of the Alter community advisory board, plans were made to tailor each aspect of the program, including the DWD guide, workshop, facilitator training, as well as the logistics of delivering the program and recruiting Black program participants. The core program team consisted of 9 Alter members, including the Program Manager, Alter Founder, Co-Founder, Grant Manager, Community Outreach Director, and 4 Community Liaisons.

### Curriculum revision

During the process of adapting the DWD comprehensive guide and workshop, we first conducted four 2-h focus group sessions with the core program team as well as volunteers and 8 Black dementia caregivers from several of Alter’s church partners. Before participating in the focus group, participants received the original DWD guide and participated in the paired workshop led by the RCI DWD Program Specialist. During the focus groups, we assessed each section of the guide to address how best to adapt content and speak to the realities of the Black caregiving experience. After all focus groups, the recorded sessions were transcribed, and the core program team analyzed conversations to determine which suggestions were feasible to apply to the guide.

We worked with a graphic designer to apply the recommendations. The title of the guide was modified to *Dealing with Dementia: A Guide for Black Caregivers*, and all pictures were changed to depict Black individuals and families. We incorporated inspirational quotes from the focus group session throughout the guide to ensure Black caregivers and community members saw themselves in the education program. Some quotes included, *“Being a caregiver may seem very hard sometimes but always remember it is definitely worth it.” “Caring for a loved one is a gift from God!” “There is help and resources available.”* In the Introduction section, we included data-driven facts and overall information about Black families faced with dementia, such as the influence of social determinants of health on Alzheimer’s disease-related ethnic and racial disparities. In the Resource Section, we included additional resources recommended by Black caregivers, such as United Way Programs and Black elder law attorneys. Outside of the Resource section in the guide, we assembled a resource packet to distribute to program participants. The packet included flyers for different dementia- and caregiver-related research studies, dementia-related and brain health pamphlets and reading material, and a breakdown of treatments to slow the progress of/address dementia-related symptoms produced by the Alzheimer’s Association (5).

Changes to the structure of the guide were also recommended and applied. For example, caregivers preferred the chapters under the section ‘General Caregiving Tips’ to be rearranged, starting with ‘The Care Team’ as chapter 1 versus chapter 3. We kept the existing fill-in handouts as they are useful tools for caregivers.

We also worked with the graphic designer to update the paired workshop slide deck to reflect culturally relevant pictures, updated information on dementia and dementia-related diseases, and visually appealing graphics and media content. Specific changes included an updated video—*A Black Family’s Battle with Alzheimer’s Disease*, page references from the guide for all 30 true and false exercises, and detailed information on caregiver burnout. The workshop maintained its original duration of 4 h.

To help reach a greater number of caregivers, we offered printed versions of the revised guide and an electronic version sent through email.

### Procedure manual

To effectively deliver the revised DWD Program, we altered RCI’s existing procedure manual (protocol) to align with the abilities of facilitators, Alter resources, and input of Alter’s advisory board that would help reach primarily Black caregivers. The protocol outlined procedures for holding virtual and in-person workshops and tips for advertising. We developed an ‘RCI-DWD’ sub-resource tab on the Alter website and advertised all upcoming workshops. The tab linked to a public-facing calendar, and as facilitators scheduled their workshop, we developed custom links to the respective registration forms for interested participants. The registration forms collected participants’ names, addresses, email addresses, and caregiving status. To accommodate participants with limited comfort with or access to technology, they could call Alter and provide the information, or facilitators could give a handwritten list of interested participants, and a core program team member would register them.

As part of the protocol, we offered $40 honoraria to caregivers who completed pre−/post-program surveys and evaluations (all had to be completed to receive the honorarium), and a $250 honorarium to facilitators for each workshop they held. We encouraged facilitators to provide food/snacks at in-person workshops, and we provided reimbursements for any costs. Mileage reimbursement was also offered to facilitators and core program team members who attended in-person workshops. At least one core program team member was present for every workshop to assist and support the facilitators, administer the DWD guides (in-person), surveys, evaluations, printed copies of the workshop slide deck so participants could take notes, and provide office supplies (pens, easel paper, markers for group discussions, and highlighters). Additionally, the core program team members set up a table to distribute the aforementioned resource packets and answer additional questions (resource packets were emailed to virtual workshop attendees).

Zoom links were set up by the core program team manager and sent to everyone who registered and the assigned facilitator. Virtual attendees received an electronic copy of the guide for the workshop and were mailed a physical copy after attending. If caregivers urgently needed the guide but could not attend a workshop, we arranged a 5–10-min individual meeting to discuss the purpose of the DWD Program and highlight the key parts of the guide. Caregivers completed RCI’s existing Individual Receipt Form and were then given a guide (either a mailed copy or an electronic version emailed, depending on preference). The Individual Receipt Form is a shortened form with the following information collected: Name, mailing address, email address, phone number, age (under 60/60 + years), Veteran status, active caregiving status, and person(s) caring for.

Alongside regular meetings with the Alter advisory board during the implementation process of the DWD_Alter_ Program, we held 1-h meetings with facilitators every 2–3 weeks. The purpose of the meetings was to bring everyone together and discuss updates/feedback about the workshops, any concerns, and to provide a space to share honest feedback on the adapted program.

### Program assessments

RCI’s existing DWD pre- and post-program surveys were used to assess the effectiveness of the updated workshops. All questionnaires included the facilitator’s name and workshop date. The pre-surveys (baseline) included demographic and caregiving background items, the Revised Scale for Caregiving Self-Efficacy (11), and the Dementia Knowledge Assessment Tool Version 2 (DKAT2) (12). We suggested minor edits to the workshop evaluation, which assessed participant acceptability using Likert-Scale responses (strongly disagree – strongly agree), that were IRB-approved and applied. For example, since the program was adapted and geared toward the needs of Black caregivers, we included the question *This workshop addressed the cultural realities of caregiving*. Other items included: *The Dealing with Dementia Guide will help me in my caregiving journey*; *The information shared in the workshop was new information to me that may improve my caregiving experience*, and an evaluation of the facilitator. The evaluation survey also included open-ended questions for participants to provide their honest feedback on the program. During in-person workshops, core program team members administered traditional printed surveys, and virtual workshop attendees completed the survey via Microsoft Forms.

### Selection of DWD program community facilitators

RCI uses a Train-the-Trainer (TTT) framework to deliver the widespread DWD program. TTT involves individuals receiving training in a given subject and instruction on how to effectively monitor and supervise others in the approach while delivering the program as intended (13). The core program team first brainstormed who should be trained as program facilitators to deliver the DWD workshops and established the following eligibility criteria: (a) 18 years of age or older, (b) self-identify as Black or African American, (c) comfortable with public speaking, (d) reside in Georgia (e) able to attend one DWD facilitator training session, and (f) able to deliver 2 at least DWD workshops (in-person or virtual). We then created an electronic screening form that included the eligibility criteria questions and asked why they wanted to become a DWD facilitator and how far they were willing to travel within Georgia. The screening form was distributed to a pre-existing pool of Alter’s advisory board members and faith community partner sites. We also promoted participation as a DWD facilitator in Alter’s monthly partner updates through MailChimp and visited several faith community partner sites in Georgia to promote this opportunity and set up numerous phone calls with Alter church ambassadors to encourage participation. Twenty Black community members were initially screened, of whom eighteen were eligible. All were affiliated with Alter’s faith-based community partners. An additional 4 eligible facilitators – friends and community members of the selected facilitators – were recruited during the pilot to support program delivery. Ten facilitators resided in rural Georgia counties (Troup, Talbot, and Dawson Counties) and 12 resided in urban settings (Fulton, Gwinnett, Newton, Dekalb, and Fayette Counties). For this project, facilitators’ and program participants’ reported zip codes were classified as either ‘urban’ or ‘rural’ using zip codes cross-referenced with the USDA’s Rural–Urban Commuting Area (RUCA) codes (14) and neighborhood classifications from the Georgia Rural Health Innovation Center (15).

Selected facilitators were oriented to the existing DWD program, receiving the original guide and participating in the paired workshop led by RCI’s DWD Program Specialist. We hosted the selected facilitators in person at the annual Alter Dementia Summit (an annual community conference focused on eradicating dementia-related disparities in the Black community through spiritual connectedness, brain health awareness, research, and community resources). The purpose of the facilitator meeting was to begin planning together how the DWD workshops would be delivered. Facilitators then participated in the DWD facilitator training, which was tailored to align with their needs. To account for facilitators’ existing work schedules, we streamlined the facilitator training by removing redundant exercises, consolidating discussion topics, and shortening its duration from 1.5 days to 4 h, while still meeting training objectives and securing approval from the RCI DWD Program Specialist. Prospective facilitators were given 3 options to attend the training (weekday evening/morning, weekend). We collaborated with the graphic designer to update the training slide deck, which included the revised family caregiver workshop slide deck and new slides on facilitator expectations and logistics. After facilitators were trained, they were incorporated into the program team and assisted with recruitment, delivery, and sustainability of DWD_Alter._ Throughout the remainder of this paper, “we” refers to both the core program team and the community facilitators.

A Facilitator “Toolkit” was developed in collaboration with the graphic designer. It included a mixture of physical and virtual materials needed to deliver the workshops. As part of the toolkit, all facilitators received: (a) printed Alter/RCI partnership press release; (b) mailed and electronic version of the adapted guide; (c) printed and emailed procedure manual; (d) printed and electronic facilitator training slide deck; (e) electronic workshop advertisement flyer templates (plug-in ready): 1 print ready & 1 social media template; (f) new family caregiver workshop slide deck: emailed, printed, and via thumb drive. During the pilot program, we held biweekly, two-hour informal Zoom meetings with facilitators to address questions, concerns, and general comments about workshop delivery.

### Participant recruitment

An extensive recruitment plan was developed in collaboration with the community facilitators to gain participation in DWD. We first collaborated with Alter’s videographer to create recruitment videos and announcements for facilitators and Georgia church partners to include in their digital bulletins.

We also shared the recruitment videos on Alter’s social media accounts regularly. We recruited participants through facilitators’ community networks, Alter’s community advisory board, and by word-of-mouth through caregivers involved in the focus groups. We shared the program in Alter’s quarterly emailed newsletters (listserv that reaches over 100 Black families affected by dementia). We coordinated with Alter’s director of community engagement to attend health fairs and community outreach events that had an influx of Black attendees, with a special focus in rural Georgia. We targeted specific Area Agencies on Aging (AAAs) in the Southern Georgia Regional Commission, Sowega Council on Aging Southwest Georgia vicinities to build partnerships and disseminate information on the revised DWD program to predominantly Black churches. Alter signed up as a vendor at Georgia festivals and community events typically frequented by Black community members, such as the wellness zone of the Annual Ice Cream Festival and the Marcus Garvey Commemoration Celebration. The pilot program was promoted at senior centers, predominantly Black faith-based communities, and agencies that serve older adults. We conducted outreach within Alter’s pre-existing pool of care partners engaged in the ‘Black Dementia Minds’ collaborative and held speaking engagements at the James M. Dixon Foundation for Alzheimer’s Research and Support Caregiver Seminar and Dementia 101 Presentation sponsored by Call for Caring Inc. and Calvin Court Senior Living Residence.

### Outcomes

Twenty-two total non-Hispanic Black community members were trained as DWD_Alter_ facilitators, comprising twenty-one females and one male. Workshops were held between June 20th, 2024, and August 31st, 2024. Fifteen facilitators held at least 1 workshop during the time frame, 13 of whom held 2 workshops, and one facilitator held 5. Twenty-seven total workshops were delivered, comprising 12 virtual, 3 hybrid, and 12 in-person sessions. Three hundred and sixty-five people registered for workshops, and 264 attended.

Four workshops were held in rural settings that took place virtually and at local Technical Colleges and libraries in Randolph and Talbot counties. The remaining workshops were held at churches and virtually in metropolitan Atlanta cities and communities. The 7 facilitators who did not hold a workshop by the end of the pilot cited unexpected medical problems, limited time, and personal schedule constraints. Of these 7, 5 were from rural communities and 2 were from urban settings. One hundred and twenty-three individuals completed the Individual Receipt Form and received a guide, but did not attend a workshop, 22 of whom received an electronic PDF version of the guide.

One hundred and eighty-four attendees completed pre-workshop surveys, and 158 completed post-workshop surveys. An assessment of participant demographics and acceptability were completed as a result of the pilot DWD_Alter_. The average age among those who reported it was 56.4 years (*n* = 177), and 79% (*n* = 177) identified as a family caregiver. The youngest attendee was 20 years of age, and the oldest was 89 years. Ninety-five percent (*n* = 178) of respondents identified as Black or African American, and 96% (*n* = 180) identified as non-Hispanic. Most attendees who reported their gender were female (*n* = 162, 87%) and employed/self-employed full-time (*n* = 81, 43%). Among those who reported their highest level of education, 4% (*n* = 37) attended some school, 20% (n = 37) completed high school/GED, and 49.5% (*n* = 91) received a bachelor’s degree or above (master’s, professional degree, doctorate). Among respondents who reported their zip codes in their personal information, 36% (*n* = 66) lived in classified rural communities. Responses to all demographic items are in Table 1.

**Table 1:** *Dealing with Dementia* pre-survey respondent demographics.

Characteristic	M [range]/*N* (%)
Age (years)	56.4 [20–89]
Gender	
(Female)	162 (87%)
(Male)	21 (11%)
(Not listed)	1 (0.5%)
Race	
(Black or African American)	178 (95%)
(White)	2 (1%)
(Asian or American)	1 (0.5%)
(Other)	3 (2%)
Ethnicity	
(Non-Hispanic/non-Latino)	180 (96%)
(Hispanic/Latino)	4 (2%)
Educational attainment	
(Some school)	6 (3%)
(High school/GED)	37 (20%)
(Some college)	35 (19%)
(Associate’ s degree)	15 (8%)
(Bachelor’s degree)	42 (23%)
(Master’s degree)	42 (23%)
(Professional/doctorate degree)	7 (4%)
Employment status	
(Employed/self-employed full-time)	81(43%)
(Employed/self-employed part-time)	12 (6%)
(Homemaker)	3 (2%)
(Retired)	66 (36%)
(Unemployed)	10 (5%)
(Other)	12 (6%)
Are you a current family caregiver?	
(Yes)	147 (79%)
(No)	37 (20%)
Are you a professional caregiver (CNA, LPN, etc.)?	
(Yes)	33 (18%)
(No)	151 (81%)
Residential zip code classification	
(Urban)	118 (64%)
(Rural)	66 (36%)

87 participants did not report age. All other demographic characteristics were missing 80 responses.

Program evaluations were completed by 201 attendees. The majority of respondents strongly agreed with the following items: *The Dealing with Dementia Guide will help me in my caregiving journey* (*n* = 159, 82%); *This workshop helped me understand how to use the guide to find the answers to my caregiving questions* (*n* = 159, 82%); *The information shared in the workshop was new information to me that may improve my caregiving experience* (*n* = 134, 69%); *I will recommend this workshop to others* and *This workshop addressed the cultural realities of caregiving* (*n* = 161, 83%). Responses to all evaluation items are shown in Table 2.

**Table 2:** *Dealing with Dementia* evaluation responses.

Question	Strongly agree	Agree	Neither agree nor disagree	Strongly disagree
The *Dealing with Dementia* Guide will help me in my caregiving journey.	159 (82%)	28 (14%)	2 (1%)	6 (3%)
This workshop helped me understand how to use the guide to find the answers to my caregiving questions.	159 (82%)	28(14%)	2 (1%)	6 (3%)
The information shared in the workshop was new information to me that may improve my caregiving experience.	134 (69%)	42 (22%)	11 (6%)	6 (3%)
Overall, the workshop was helpful to me.	163 (84%)	25 (13%)	1 (0.5%)	6 (3%)
I will recommend this workshop to other caregivers.	175 (90%)	12 (6%)	2 (1%)	6 (3%)
This workshop addressed the cultural realities of caregiving.	161 (83%)	26 (13%)	7 (4%)	1 (0.5%)

Responses	About right	Too long	Too short	
Did you feel the length of the workshop was…	179 (92%)	12 (6%)	4 (2%)	

## Discussion

### Program implications and constraints

Approximately 375,000 family caregivers in the U.S. support someone living with dementia or dementia (Alzheimer’s Association, 2024). As the late Rosalynn Carter aptly stated, “*There are only four kinds of people in the world*—*those who have been caregivers, those who are currently caregivers, those who will be caregivers, and those who will need caregivers* (8).” In this context, the adapted DWD program reached 387 individuals, whether through receiving the guide with a brief overview of the information or by participating in the paired workshop. While many participants were active caregivers, the inclusion of former caregivers and individuals anticipating future caregiving roles meant that vital information reached beyond immediate needs, offering preparation and support for those on caregiving journeys or assisting someone in their circle.

Applying CBPR principles—centering the community through shared decision-making, utilizing local resources, and iteratively gathering feedback—enhanced the cultural relevance and acceptability of the program. Partnering with the community strengthened the development and implementation process and fostered trust and ownership. The high volume of “strongly agree” responses and overwhelming positive open-ended feedback in participant evaluations validated the value of culturally responsive approaches. Research has shown that culturally tailored education fosters greater engagement and helps learners retain information more effectively (16, 17).

Despite its successes, the pilot DWD_Alter_ faced key limitations. Because the limited grant period allowed only 2.5 months for facilitators to deliver the workshops, pre- and post-data collection was limited, preventing meaningful comparisons with findings from earlier evaluations of the DWD program.

Additionally, the short timeline prevented 7 facilitators from hosting a DWD workshop before the end of the grant period due to scheduling and availability constraints. Additionally, a program participant noted that using “Black” in the title seemed unnecessary if the emphasis on Black caregivers was not sustained through content. While the workshop, guide, and accompanying imagery referenced Black families, the core content remained broadly applicable, and some felt it insufficiently centered the Black caregiving experience, despite its name, *Dealing with Dementia: A Guide for Black Caregivers*. Although components of the program were adapted based on input from Black faith and community members, incorporating specific frameworks and caregiver narratives to explicitly and intricately guide the development of culturally tailored content could have further enhanced the relevance and impact of the information. With additional time and resources, the tailoring process could have been more comprehensive and better aligned with culturally specific caregiving models.

Another limitation was the program’s limited reach in rural communities, a priority we had hoped to address more fully. Of the 7 program facilitators who did not deliver workshops by the deadline, 5 were based in rural communities (half of the total rural-based program facilitators), representing a significant shortfall. Consequently, many rural Black caregivers may not have had the opportunity to participate. This shortfall highlights a broader issue: the need to equitable access to resources in Black rural communities, which remained a shortcoming of our DWD_Alter_. If we had supported these facilitators in organizing participation and spaces for the program, we could have substituted other facilitators to ensure the education was still provided. In addition, among the 184 workshop attendees who completed pre-program surveys, only 66 resided in rural communities—a lower proportion than expected based on outreach efforts. This shortfall is especially concerning given the well-documented lack of access to formal caregiving support and dementia education in rural communities (18). Structural barriers such as transportation challenges, healthcare workforce shortages, and lower general awareness of Alzheimer’s disease and related dementias often leave rural caregivers underserved (19). Black caregivers in rural communities are particularly vulnerable to “falling through the cracks” of existing support systems, despite facing disproportionate caregiving burdens and limited culturally competent care options (1). Meeting caregivers “where they are” remains one of the most pressing challenges (18). This includes ensuring awareness of available resources and offering services in trusted community spaces. Encouragingly, two program facilitators responded to the limited rural participation in the DWD program by independently continuing workshop delivery in LaGrange and Box Springs, Georgia, well beyond the grant period. In recognition of these ongoing needs, the core program team has begun further developing/strengthening partnerships with rural Black churches to enhance outreach and deepen connections with local caregivers. Strengthening these place-based partnerships is a critical next step in expanding the reach and long-term sustainability of culturally tailored caregiver support.

### Program sustainability

The development and delivery of the adapted DWD Program came with its fair share of challenges. To ensure the lessons learned were sustained, a formal Sustainability plan was co-developed with the program facilitators to ensure that resources spent on the DWD Program were not lost. As a result, DWD_Alter_ was integrated into the Alter framework as an ongoing support and education resource. Today, Alter’s resources reach more than 90 faith-based partnerships nationwide, expanding the program’s reach and positioning DWD_Alter_ to have a lasting impact on Black communities across the country. Program sustainability is an important component of public health practice, ensuring that effective interventions continue to benefit communities beyond initial funding periods (20). Sustainable programs are more likely to maintain positive health outcomes, build long-term community trust, and adapt to evolving needs over time. Without sustainability planning, even well-designed initiatives risk losing momentum or failing to address health disparities in a lasting way. Embedding sustainability strategies—such as community partnerships, capacity building, and policy integration—can help institutionalize public health gains and promote health equity (20).

In efforts to encourage shared responsibilities, a sustainability planning meeting was held in September 2024, with 10 community facilitators and 3 core program team members to discuss actions to support this project for a long-term period. During this planning meeting, attendees discussed their experience as facilitators and holding DWD workshops in their communities. Considering the discussion and feedback received throughout the project, the following steps will be implemented to sustain Alter’s and the community’s effort to deliver the adapted DWD workshop and guide to Black caregivers in Georgia.

Alter will establish a partnership with 8 libraries in South Fulton for the community facilitators to host workshops. Under the guidance of the program core team, current community facilitators will continue to organize and deliver workshops in their respective communities using the tools they were provided during the project period. Facilitators will continue to be responsible for securing workshop locations and videoconferencing platforms to hold the workshop. Facilitators will also continue to be responsible for the advertisement of workshops and submitting requests to Alter’s Director of Community Engagement to advertise the workshops on Alter’s event page.

While initial outcomes focused on participant demographics and satisfaction (acceptability), more robust analysis is needed to evaluate effectiveness, including changes in caregiving self-efficacy and dementia knowledge before and after program participation. Collaboration with the Rosalynn Carter Institute’s Data Manager to conduct statistical analysis and assess these outcomes is underway.

## Data Availability

The datasets presented in this article are not readily available because the data outlined in the outcomes were preliminary evaluative data. Although collected, pre-post data has not yet undergone in-depth analysis with a biostatistician. Requests to access the datasets should be directed to Karah Alexander, karah.lynea.alexander@emory.edu.
